# Comparison of Efficacy and Safety of Low-Dose Versus High-Dose Dexamethasone in Hospitalized COVID-19 Patients: A Meta-Analysis

**DOI:** 10.7759/cureus.33884

**Published:** 2023-01-17

**Authors:** Muhammad Daniyal Waheed, Aimen Shaikh, Shazaf M Sidhu, Salwan Ahmad, Tehreem Sikander, Aizaz R Chaudhry, Izza Iftikhar, Tanveer Ahamad Shaik

**Affiliations:** 1 Internal Medicine, Foundation University Medical College, Islamabad, PAK; 2 Medicine, Foundation University Medical College, Islamabad, PAK; 3 Medicine, Jinnah Medical and Dental College, Karachi, PAK; 4 Internal Medicine, Shifa College of Medicine, Shifa Tameer-e-Millat University, Islamabad, PAK; 5 Internal Medicine, Jinnah Medical and Dental College, Karachi, PAK; 6 Emergency Department, Bahawal Victoria Hospital, Bahawalpur, PAK; 7 Internal Medicine, Quaid-e-Azam Medical College, Bahawalpur, PAK; 8 Cardiovascular Medicine, University of Louisville School of Medicine, Louisville, USA

**Keywords:** sars-cov-2, corticosteroids, intensive care, coronavirus disease 2019, covid-19, low dose, high dose, meta-analysis, dexamethasone

## Abstract

The aim of this study is to compare the efficacy and safety of low-dose and high-dose dexamethasone in hospitalized coronavirus disease 2019 (COVID-19) patients. The current meta-analysis was conducted in compliance with Preferred Reporting Items for Systematic Reviews and Meta-Analyses (PRISMA) guidelines. A comprehensive literature search was carried out using PubMed, the Cochrane Central Register of Controlled Trials (CENTRAL), and Embase. Outcomes assessed in the current meta-analysis included 28-day mortality, intensive care unit (ICU) admission, mechanical ventilation, length of ICU admission (days), and length of hospital stay (days). For safety, we compared hypoglycemia and the incidence of infection between the high-dose dexamethasone group and the low-dose dexamethasone group. A total of four studies fulfilled the inclusion criteria and were included in this meta-analysis. No significant difference was found between the two groups in terms of ICU admission (risk ratio (RR): 0.72, 95% confidence interval (CI): 0.41-1.28, p-value: 0.27), length of stay in ICU in days (mean difference (MD): -0.05, 95%CI: -3.96-3.87, p-value: 0.98, I-square: 94%), length of hospital stay in days (MD: -0.94, 95%CI: -1.94-0.06, p-value: 0.07), need of mechanical ventilation (RR: 0.72, 95%CI: 0.36-1.48, p-value: 0.38), and 28-day mortality (RR: 0.90, 95% CI: 0.50-1.64, p-value: 0.74). The current study showed that higher doses of dexamethasone failed to enhance efficacy compared to low-dose dexamethasone. Thus, based on the findings of this meta-analysis, low-dose dexamethasone can be recommended for these patients.

## Introduction and background

Patients with critical coronavirus disease 2019 (COVID-19) are characterized by hypoxemia and pulmonary inflammation that often leads to the use of mechanical ventilation and high-flow oxygen [1]. The initial symptoms of COVID-19 are sore throat, headache, malaise, dyspnea, fever, and other influenza-related symptoms [2]. However, organ failure and acute respiratory distress syndrome (ARDS) are common in severe and critical cases [3]. Since the beginning of the epidemic, researchers have been vying for the best COVID-19 treatments. However, not many therapy alternatives are accessible right now [4]. Drugs such as interferons, protease inhibitors, and hydroxychloroquine were considered effective as per the initial experience. Still, they were withdrawn later from protocols because of the risk of adverse effects and lack of efficacy [5-6].

A recent meta-analysis by Sterne et al. showed improved outcomes in patients with moderate or severe COVID-19 treated with corticosteroids [7]. However, the doses (low versus high doses) and corticosteroid types used in these clinical trials were different. World Health Organization (WHO) recommended corticosteroids for patients with severe and critical COVID-19. However, the dosages used among patients with COVID-19 varied across different countries and hospitals [8]. Dexamethasone has wide effects on adaptive and innate immunity. Adaptive immunity is integral to COVID-19 immunopathology as the beginning of ARDS coincides temporally with the emergence of a particular antibody against severe acute respiratory syndrome coronavirus 2 (SARS-CoV-2) [9].

Studies showed that overwhelming inflammation is linked with critical and severe cases of COVID-19 [10]. A higher dose of dexamethasone has been utilized for ARDS not related to COVID-19 [11]. Pharmacodynamic investigations have found a dose-dependent glucocorticoid impact on biomarkers of glucocorticoid receptor agonism [12]. However, increased doses of corticosteroids enhance the risk of adverse events, especially infections and hyperglycemia [11]. Recently, few clinical trials have been conducted that compared the efficacy and safety of dexamethasone in patients hospitalized with COVID-19 as, currently, it is unclear what would be the optimal dose of dexamethasone. The aim of this meta-analysis is to compare the efficacy and safety of low-dose and high-dose dexamethasone in hospitalized COVID-19 patients.

## Review

Methodology

The current meta-analysis followed the guidelines of the Preferred Reporting Items for Systematic Reviews and Meta-Analyses (PRISMA) statement.

Search Strategy

A comprehensive literature search was performed by two authors (AS and SA) independently using PubMed, the Cochrane Central Register of Controlled Trials (CENTRAL), and Embase. No restrictions were placed on language and year of publication. The search terms used to identify relevant studies included “COVID-19”, “dexamethasone”, “high dose”, “low dose”, and “efficacy”.

Study Selection and Data Extraction

In the first stage, title and abstract screening of all articles were conducted by two authors (MW and TS) independently to assess eligibility criteria. In the second stage, full texts of all eligible articles were retrieved and screening was done according to the inclusion and exclusion criteria. Any disagreement during the study selection process was resolved through discussion. For data extraction, a data collection sheet was designed in Microsoft Excel (Microsoft Corporation, Redmond, Washington, United States). Data extraction was performed by two authors (AS and II) independently. Data extracted included author name, year of publication, study groups, sample size, dexamethasone dosage, patients characteristics (mean age in years and number of males), and measured outcomes.

Eligibility Criteria

We included all randomized control trials (RCTs) comparing high-dose and low-dose dexamethasone in hospitalized COVID-19 adult patients. We excluded observational studies, non-randomized trials, case series, case reports, and reviews. The inclusion criteria for low-dose dexamethasone was ≤ 10 mg/day, while any dose higher than low-dose dexamethasone was considered high-dose dexamethasone.

Outcome and Risk-of-Bias Assessment

We assessed 28-day mortality, intensive care unit (ICU) admission, mechanical ventilation, length of ICU admission (days), and length of hospital stay (days) in the present meta-analysis as efficacy outcomes. For safety, we compared hypoglycaemia and the incidence of infection between the high-dose dexamethasone group and the low-dose dexamethasone group.

Risk-of-bias assessment was carried out using the Cochrane Risk-of-Bias Assessment Tool by two authors (SA and AC) who independently included an assessment of performance bias, selection bias, attrition bias, detection, bias, reporting bias, and other potential bias sources. Any disagreement during the risk-of-bias assessment was resolved through discussion.

Statistical Analysis

Review Manager, version 5.4.0 (The Nordic Cochrane Center, The Cochrane Collaboration, Copenhagen, Denmark) was used for data analysis. Continuous outcomes were presented as mean difference (MD) along with 95% confidence interval (CI), while categorical outcomes were presented as risk ratio (RR) with their 95%CI. A p-value less than 0.05 were considered significant. Statistical heterogeneity was assessed using I-square statistics. A fixed-effect model was used if heterogeneity was less than 50% in any of the outcomes; otherwise, the random-effect model was used.

Results

Literature Search

**Figure 1:**
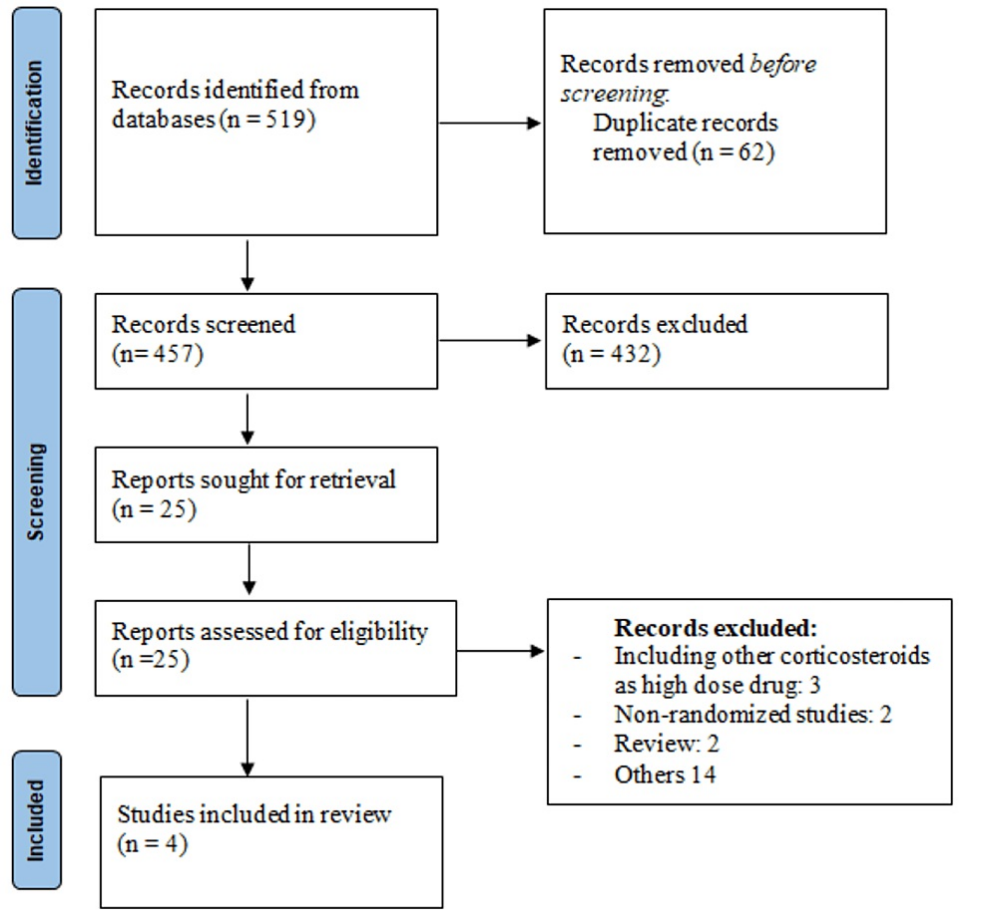
PRISMA flowchart of selection of studies PRISMA: Preferred Reporting Items for Systematic Reviews and Meta-Analyses

A total of 519 records were selected by online database searching, with 62 records being duplicates; 432 articles were removed based on the abstract and title screening. The full text of 25 potential articles was retrieved for inclusion and exclusion criteria. A total of four studies were included in the current meta-analysis based on the inclusion and exclusion criteria. The process of selection of studies is shown in Figure 1.

Basis Characteristics and Quality Assessment

**Figure 2:**
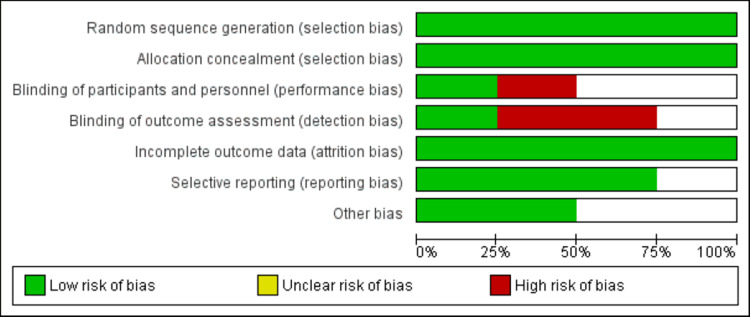
Risk-of-bias assessment

The characteristics of the four included RCTs are shown in Table 1. The studies included 1383 patients (689 patients received low-dose dexamethasone and 693 patients high-dose dexamethasone) including three single-center studies and one multi-center study. The mean age of included studies ranged from 57 years to 64.5 years and most participants were males. Figure 2 shows the results of the quality assessment of the included studies. Among all included studies, two studies were single-blind, while one was unblinded.

**Table 1:** Characteristics of included studies

Author Name	Year	Setting	Group	Dose	Sample Size	Mean age (Years)	Males (%)
Munch et al. [13]	2021	Multi-center	Low dose	6 mg	485	64.5	68.94%
			High dose	12 mg	497		
Taboada et al. [14]	2021	Single-center	Low dose	6 mg	102	64.3	61.80%
			High dose	20 mg for first five days and 10 mg daily for last five days	98		
Toroghi et al. [15]	2022	Single-center	Low dose	8 mg	47	57.5	63.44%
			High dose	24 mg	46		
Wu et al. [16]	2022	Single-center	Low dose	6 mg	55	57	54.20%
			High dose	20 mg for first five days and 10 mg daily for last five days	52		

Outcome Meta-analysis

**Figure 3:**
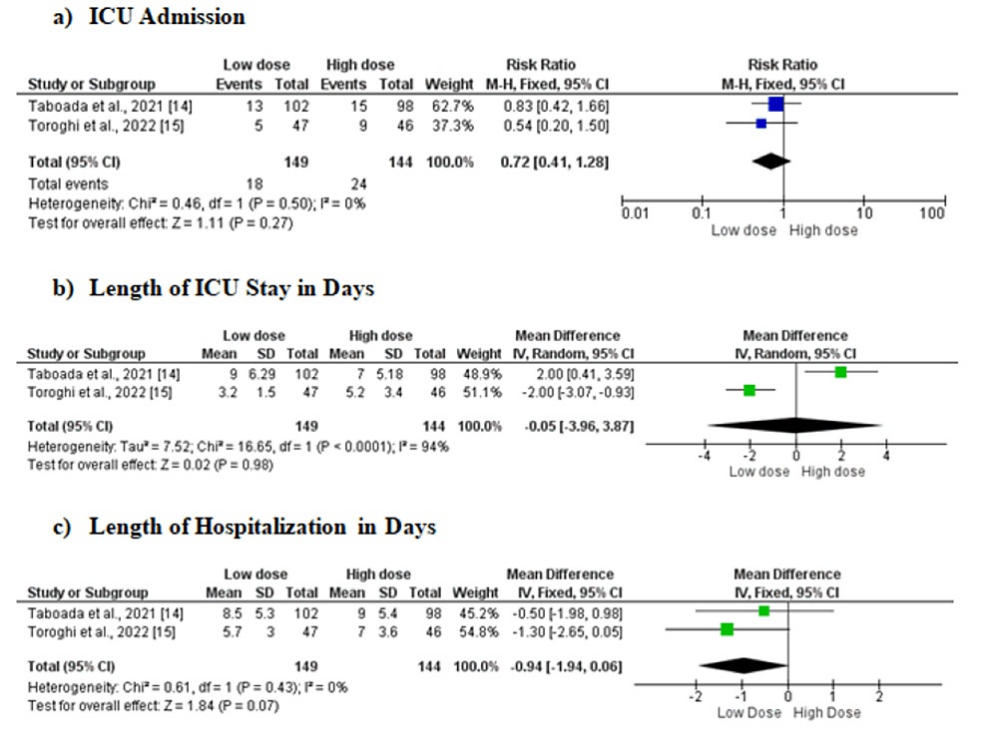
Forest plot comparing the effect of low-dose versus high-dose dexamethasone on (a) ICU admission, (b) length of ICU stay, and (c) length of hospitalization M-H: Mantel-Haenszel

Two RCTs [14,15] compared the incidence of ICU admission between 149 low-dose dexamethasone patients and 144 high-dose dexamethasone patients. No significant difference was found between the two groups in terms of ICU admission (RR: 0.72, 95%CI: 0.41-1.28, p-value: 0.27). Low heterogeneity was found among study results (I-square: 0%). For those patients who were admitted to ICU, no significant difference in length of stay in the ICU in days was found between the two study groups (MD: -0.05, 95%CI: -3.96-3.87, p-value: 0.98, I-square: 94%). Two studies evaluated the effects of the dose of dexamethasone on the length of hospital stay in days. Overall analysis showed that the mean length of hospital stay was lower in patients randomized to the low-dose dexamethasone group but the difference was statistically insignificant (MD: -0.94, 95%CI: -1.94-0.06, p-value: 0.07) with no heterogeneity (I-square: 0%) (Figure 3).

**Figure 4:**
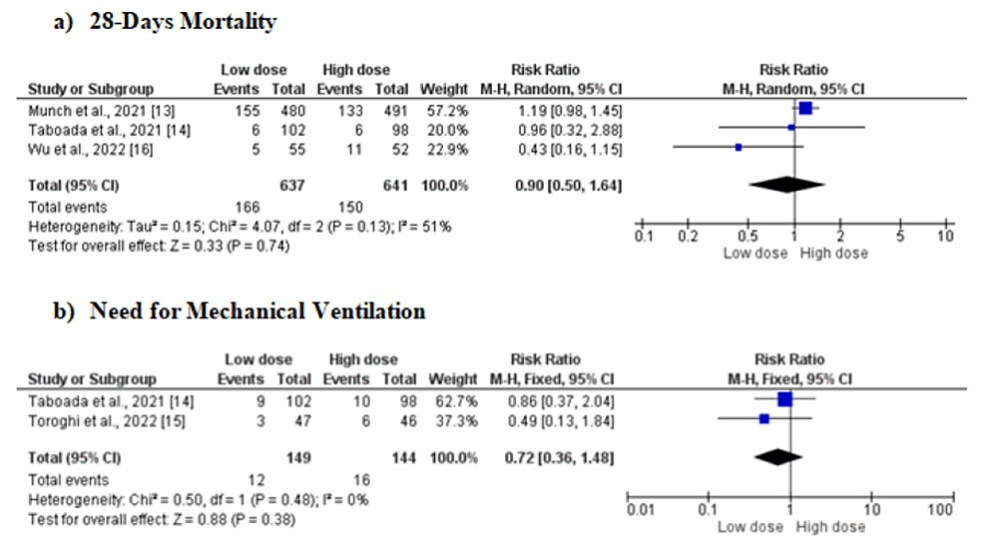
Forest plot comparing the effect of low-dose versus high-dose dexamethasone on (a) 28-day mortality and (b) need for mechanical ventilation M-H: Mantel-Haenszel

Three studies [13-16] showed that there is no significant difference in 28-day mortality between the two groups (RR: 0.90, 95%CI: 0.50-1.64, p-value: 0.74). Moderate heterogeneity was found among the study results (I-square: 51%). Two RCTs reported mechanical ventilation in 149 low-dose dexamethasone patients and 144 high-dose dexamethasone patients. In this meta-analysis, no significant difference was reported in terms of the incidence of mechanical ventilation between the two study arms (RR: 0.72, 95%CI: 0.36-1.48, p-value: 0.38). No heterogeneity was reported between the study results (I-square: 0%) (Figure 4).

**Figure 5:**
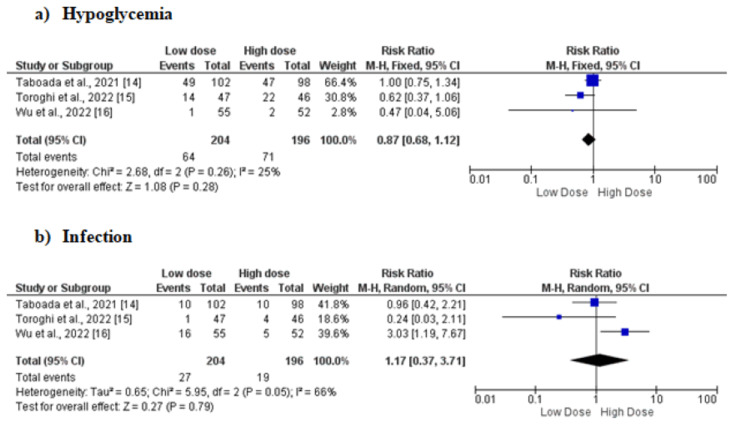
Forest plot comparing the effect of low-dose versus high-dose dexamethasone on (a) risk of hypoglycemia and (b) risk of infection M-H: Mantel-Haenszel

Three studies [14-16] assessed the risk of infection and hypoglycemia in patients receiving low-dose and high-dose dexamethasone. No significant difference was found in the incidence of hypoglycemia and incidence of infection between the two study arms as shown in Figure 5.

Discussion

The current meta-analysis compared the safety and efficacy of high-dose and low-dose dexamethasone in hospitalized COVID-19 patients. To the best of our knowledge, this is the first meta-analysis comparing the safety and efficacy of low-dose versus high-dose dexamethasone in hospitalized COVID-19 patients. No significant difference was reported in ICU admission, length of ICU stay, need for mechanical ventilation, length of hospital stay, 28-day mortality rate, and incidence of infection rate and hyperglycemia between the patients randomized in low-dose and randomized high-dose dexamethasone groups.

The findings of our meta-analysis are consistent with the meta-analysis conducted by Tan et al. [8] and Cano et al. [17] comparing high-dose corticosteroids and low-dose corticosteroids. Our meta-analysis has focused on low-dose dexamethasone and high-dose dexamethasone. As a small number of studies compared low-dose and high-dose dexamethasone and the non-superiority of high-dose dexamethasone in the current meta-analysis, we cannot claim that high-dose dexamethasone is more effective. Thus, future studies need to be conducted including a larger sample size to evaluate the safety and efficacy of various doses of dexamethasone in the management of hospitalized COVID-19 patients. 

Low-dose corticosteroids like dexamethasone commonly are used in surgery for minimizing vomiting and nausea and as an adjunct to part of the multimodal analgesia. The corticosteroids used for surgery have side effects that depend on the dosage, including an increased risk of hyperglycemia, surgical site infections, myocardial infarctions, and mortality [18]. We assessed the rate of infection and hyperglycemia and did not find any significant difference between low-dose and high-dose dexamethasone patients. In this trial, new infections, and hyperglycemia were comparable in both groups despite the possibility that increased doses of corticosteroids would be linked with more complications. This is consistent with earlier studies that did not find an increased risk of adverse events with corticosteroids in patients with ARDS with or without COVID-19 [19,20].

Despite corticosteroids' beneficial anti-inflammatory effects, there is conflicting evidence about their safety when used in patients with COVID-19 [21,22]. The Coronavirus Dexamethasone (CoDEX) clinical trial did not show a significant difference in terms of risk of bacteremia, new infection, or any other insulin use for hyperglycemia in the 20 mg dexamethasone group compared to a standard care group [23]. The meta-analysis showed that high-dose dexamethasone was not associated with an increased risk of adverse events. Because of the limited availability of data, we only extracted data for the incidence of infection rate and hyperglycemia between the low-dose and high-dose dexamethasone groups. The study conducted by Mondero et al. reported that moderate to high-dose corticosteroids (1 mg/kg/d methylprednisolone or 0.12 mg/kg/d dexamethasone) was not linked with an increased risk of infection or medical complications [19].

Certain steps need to be taken to understand the impacts of dexamethasone in hospitalized COVID-19 patients. Firstly, more studies on endogenous immune response and viral clearance after and before the administration of dexamethasone can provide insight into the possible benefits and harms of dexamethasone. Deficiencies in dysregulated endogenous proinflammatory responses and interferon responses [24], which could be assessed in circulating blood samples both before and following dexamethasone therapy, have been linked to COVID-19 mortality.

The current meta-analysis has certain limitations. Firstly, the number of included studies is only four and due to this, we did not assess publication bias. Secondly, the outcomes of long-term follow-up for low-dose and high-dose dexamethasone COVID-19 survivors are required in order to determine any complication of dexamethasone in the relapse of pneumonia or ARDS. In addition, we were not able to perform subgroup analysis based on the COVID-19 severity because of limited data available.

## Conclusions

To the best of our knowledge, this is the first meta-analysis comparing the safety and efficacy of different doses of dexamethasone in hospitalized COVID-19 patients. The current meta-analysis found no significant difference in ICU admission, length of ICU stay, length of hospital stay, 28-day mortality, need for mechanical ventilation, and adverse events. The current study showed that higher doses of dexamethasone failed to enhance efficacy compared to low-dose dexamethasone. Thus, based on the findings of this meta-analysis, low-dose dexamethasone can be recommended for these patients. In the future, larger studies need to be conducted including a larger sample size to assess the optimum dose of dexamethasone in COVID-19 hospitalized patients. 
